# Genetic Relationships in the Toxin-Producing Fungal Endophyte, *Alternaria* *oxytropis* Using Polyketide Synthase and Non-Ribosomal Peptide Synthase Genes

**DOI:** 10.3390/jof7070538

**Published:** 2021-07-06

**Authors:** Rebecca Creamer, Deana Baucom Hille, Marwa Neyaz, Tesneem Nusayr, Christopher L. Schardl, Daniel Cook

**Affiliations:** 1 Department of Entomology, Plant Pathology and Weed Science, New Mexico State University, Las Cruces, NM 88003, USA; dlhille2017@gmail.com (D.B.H.); marwane@nmsu.edu (M.N.); 2 Arts and Science Biology Department, University of Houston, Victoria, TX 77901, USA; tnusayr@gmail.com; 3 Department of Plant Pathology, University of Kentucky, Lexington, KY 40508, USA; schardl@uky.edu; 4 Poisonous Plant Laboratory, US Department of Agriculture, Logan, UT 84341, USA; Daniel.cook@ars.usda.gov

**Keywords:** *Alternaria* sect. *Undifilum*, polyketide synthase, secondary metabolite, nonribosomal peptide synthase, swainsonine biosynthesis

## Abstract

The legume *Oxytropis sericea* hosts a fungal endophyte, *Alternaria* *oxytropis*, which produces secondary metabolites (SM), including the toxin swainsonine. Polyketide synthase (PKS) and non-ribosomal peptide synthase (NRPS) enzymes are associated with biosynthesis of fungal SM. To better understand the origins of the SM, an unannotated genome of *A. oxytropis* was assessed for protein sequences similar to known PKS and NRPS enzymes of fungi. Contigs exhibiting identity with known genes were analyzed at nucleotide and protein levels using available databases. Software were used to identify PKS and NRPS domains and predict identity and function. Confirmation of sequence for selected gene sequences was accomplished using PCR. Thirteen PKS, 5 NRPS, and 4 PKS-NRPS hybrids were identified and characterized with functions including swainsonine and melanin biosynthesis. Phylogenetic relationships among closest amino acid matches with *Alternaria* spp. were identified for seven highly conserved PKS and NRPS, including melanin synthesis. Three PKS and NRPS were most closely related to other fungi within the Pleosporaceae family, while five PKS and PKS-NRPS were closely related to fungi in the Pleosporales order. However, seven PKS and PKS-NRPS showed no identity with fungi in the Pleosporales or the class Dothideomycetes, suggesting a different evolutionary origin for those genes.

## 1. Introduction

The endophytic fungus *Alternaria* (section *Undifilum*) *oxytropis* (Q. Wang, Nagao and Kakish.) Woudenb and Crous produces many secondary metabolites, including swainsonine, a compound toxic to mammals. This and related *Alternaria* sect. *Undifilum* spp. (family Pleosporaceae, order Pleosporales, class Dothideomycetes) are found within locoweeds, *Astragalus* and *Oxytropis* species that contain swainsonine. When these plants are ingested by mammals such as cattle, horses, sheep, and goats, normal cellular function is disrupted resulting in locoism disease ([1,2]. Other fungi, including *Slafractonia leguminicola* (order Pleosporales), *Metarhizium anisopliae* (class Sordariomycetes), members of the Arthodermataceae, and a yet undescribed Chaetothyrialean endophyte from *Ipomoea carnea* also produce swainsonine [3,4,5]. The biosynthetic pathway for swainsonine has been partially characterized that includes a gene cluster SWN which include a hybrid nonribosomal peptide synthetase (NRPS)-reducing ketide synthase *swnK* that was demonstrated to be essential for swainsonine biosynthesis in *Metarhizium robertsii* using a knockout of the gene through homologous gene replacement that lacked swainsonine production and complementation that restored its presence [6]. This work identified a SWN gene cluster associated with swainsonine biosynthesis in *Alternaria oxytropis, S. leguminicola, Arthroderma* and the Chaeothyriales fungus associated with *Ipomoea carnea*, and showed that the order of the different domains within *swnK* was identical among all the swainsonine-producing fungi, including the Chaetothyriales endophyte from *Ipomoea carnea*, *Metarhizium* spp., and the dermatophytes. Noor et al. [7] demonstrated that the KS portion of PKS-NRPS is highly conserved among all swainsonine-producing *Alternaria* spp., but differed slightly between plant pathogens and nonpathogens.

Many fungi produce specialized secondary metabolites (SM) for virulence, defense, or communication [8]. Biosynthetic pathways of many SMs include steps catalyzed by multifunctional polyketide synthase (PKS) enzymes. These enzymes direct the structure and biosynthesis of the compounds produced and contain multiple domains leading to a high diversity of SMs [9]. The high diversity in SMs obscures understanding of how SM pathways have evolved in fungi. In addition to duplications, domain shuffling, neofunctionalization, and subfunctionalization, some SM gene clusters are suggested to be transferred between diverse fungi by horizontal (lateral) gene transfer (HGT) [8].

Genes associated with toxin biosynthetic pathways in fungi have been found to encode PKSs and NRPSs [10,11]. They are often clustered with genes encoding other steps in the same pathway and can be identified from genomes using prediction software based on PKS domains [12]. Different functional types of PKSs can be identified from sequence and domain structure. Fungi can have type I PKSs, of which there can be highly reducing (HR), non-reducing (NR), or partially reducing (PR) categories, all of which begin with simple carboxylic acids and are iterative [13]. In addition, fungi have NRPSs that use amino acids as starter units for building compounds. Hybrids involving both PKS and NRPS domains have also been identified in fungi and catalyze production of many different polyketide and amino acid hybrid compounds [14].

Genome mining of toxin-producing *Fusarium* spp. (class Sordariomycetes) for SM genes has revealed 30, 32, 28, and 26 PKS and NRPS gene clusters for *F. graminearum*, *F. verticillioides*, *F. oxysporum*, and *F. solani*, respectively [15]. Examining the genomes of *Aspergillus* spp. has also revealed a range of genes. Using multiple software programs along with manual annotations, total clusters (including NRPS-like enzymes) predicted for *A. nidulans*, *A. fumigatus*, *A. niger*, and *A. oryzae* are 71, 39, 81, and 75, respectively [16]. SM genes have also been identified through genome analysis for the endophytic fungus *Pestalotiopsis fici* [17]. In *P. fici*, 27 PKSs, 12 NRPSs, and five PKS-NRPS hybrids have been identified [18]. Perhaps due to their growth habits, endophytes may have smaller numbers of PKSs compared to *Fusarium* and *Aspergillus*.

To better understand the origins of PKS, NRPS, and PKS-NRPS genes, we investigated their presence in the swainsonine-producing fungus, *Alternaria oxytropis*. We sought to use genomic analyses to identify PKSs, NRPSs, and hybrid PKS-NRPSs, and predict their structures and functions. The evolutionary relationships among these genes suggest diverse origins for SMs.

## 2. Materials and Methods

The genome of *A. oxytropis* isolated from *Oxytropis sericea* collected from Raft River, UT in 1979 was sequenced using Illumina MiSeq with 250 bp paired-end reads on a 400 bp library in Advanced Genetics Technology Center at the University of Kentucky as reported in Cook et al. [6]. Assembly was done using CLC Genomics Workbench 8.0.2 (Qiagen, Germantown, MD, USA). An unannotated, partially assembled genome (due to high number of repeated sequence) was obtained. For this work, the partially assembled genome was made into a searchable database in Geneious v. R8 (Geneious, CA, USA) [19]. To identify contigs containing PKS genes, several known nucleotide sequences were used to search the database using the parameters of a discontiguous megablast. Thirty nucleotide sequences of fungal PKS genes were used as query for matching contigs in the *A*. *oxytropis* genome including three obtained from the biosystems database at the National Center for Biotechnology Information (NCBI) and 27 from Clustermine 360, the database of microbial PKS/NRPS [20,21].

Each contig that was identified from the initial search was analyzed for nucleotide sequence similarity using a discontiguous megablast search function of the nucleotide redundant database of NCBI. Contigs containing partial sequence of PKS genes were completed by searching the *A. oxytropis* genome for similar matches and manual assembly of contigs within the Geneious software. Maximum parsimony analyses of *A. oxytropis* PKS gene contigs along with Clustermine360 fungal PKS genes was used to identify duplicate sequences and determine PKS types (Paup, 1000 bootstrap). All contigs containing *Pks-*like genes were characterized through BLASTn, BLASTp, Smartblast, and Conserved Domain Database searches of the NCBI database along with analysis through antibiotics and Secondary Metabolite Analysis SHell (antiSmash) v. 3.0 and Secondary Metabolites Unknown Regions Finder (SMURF) [22].

Various programs were used to analyze NRPS and PKS genes and domains. AntiSmash was also used to predict NRPS, PKS and secondary metabolites [23,24]. Natural Product Domain Seeker (NaPDoS) was used to identify and predict KS and C domains [25], and Non-ribosomal Peptide Synthase Substrate Predictor (NRPSsp) was used to predict NRPS substrates [26]. InterProScan was used to form predictive models [27], and Secondary Metabolite Prediction and Identification (SeMPI) was used to predict and identify pipelines for PKS and NRPS [28].

Confirmation of sequence for selected sequences was accomplished using PCR and by comparison with a second partial genome sequence of *A. oxytropis*. Primers were designed based on the obtained sequences in Geneious, and PCR was performed as previously reported [29]. All sequences were deposited into Genbank and accession numbers are listed in Table 1. Closest matches by BLASTp were based on percentage identity for sequences with query coverage greater that 90%.

The highest seven matches for each amino acid sequence using BLASTp were used for generating phylogenetic trees. Sequences were aligned with MUSCLE using Geneious 10.0.9 software and trees were generated using PAUP and maximum parsimony with 100 replicates. Outgroups are marked on each tree and were the sixth closest matches for each tree.

## 3. Results

### 3.1. Phylogenetic Framework

Twenty-two PKS and NRPS genes, were identified from the *A. oxytropis* genome; 13 were identified as type I PKSs, five as NRPSs, and four as hybrid PKS-NRPSs. Each gene has been named as the functional type of gene followed by the original contig number from the genome search (Table 1 and Table 2). Closest matches by BLASTp were based ranged from a low of 46% to a high of 95% identity for sequences with 97–100% query coverage. All but two of the samples had a query coverage of 99 or 100%.

Several different domains were identified among the PKSs using software to predict domains including: ketoacyl synthase (PKS), acyl transferase (AT), dehydratase (DH), methyl transferase (MT), acyl carrier protein (ACP), enoyl reductase (ER), and ketoreductase (KR). Among the NRPSs the following were identified: adenylation (A), terminal reductase (TD), condensation (C), peptidyl carrier protein (PCP) (Supplemental Figure S1). Importantly, not all domains were identified in each gene of a particular PKS or NRPS.

### 3.2. PKS-NRPS Hybrids

Four hybrid PKS-NRPSs were identified, all were similar to a genus in the Ascomycota, two associated with plants and the other two associated with insects or animals, and only one has an established function (swainsonine biosynthesis) (Table 1).

**Figure 1:**
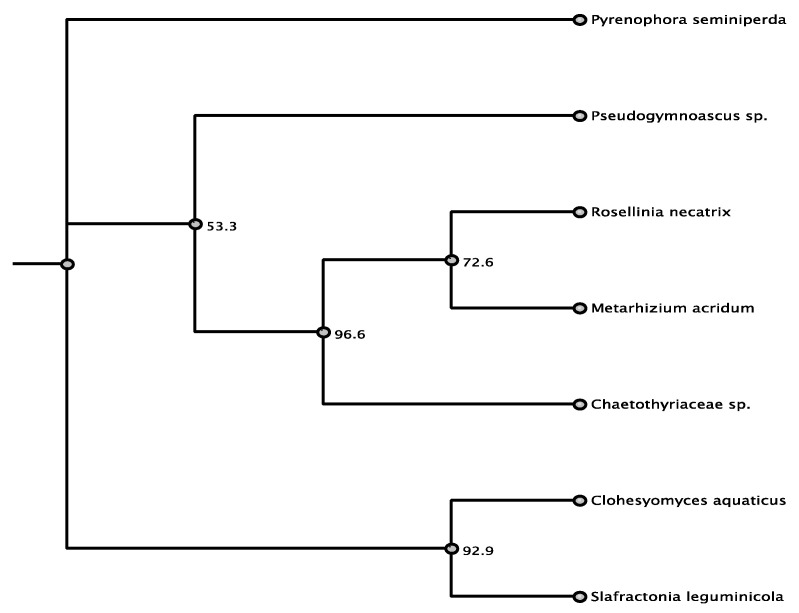
Maximum parsimony tree (MUSCLE alignment and 1000 replicates). *Pseudogymnoascus* sp. was used as the outgroup. Fungi in tree are pblast results of 58882 sequence.

The presence of *swnK* was verified for PKS-NRPS 58882 (Figure S1). Secondary metabolite product prediction also verified that PKS-NRPS 58882 was likely to produce swainsonine and establishing that the software was effective (Table 2). A BLASTn search verified that this PKS-NRPS was most similar to the swainsonine-producing fungi *Slafractonia leguminicola*, a Chaetothyriaceae, and several *Metarhizium* sp. Alignment identity for nucleic acid was 74%, with the *swnK* gene of *S. leguminicola*, 70% with the swainsonine biosynthesis gene cluster of Chaetothyriaceae, and 69% with the same cluster in *M. robertsii*. A BLASTp search revealed matches to a polyketide synthase from *Pyrenophora seminiperda* (Pleosporaceae, Pleosporales, Dothideomycetes) (81% amino acid identity), *Slafractonia leguminicola* (Pleosporales) *SwnK* (73%), a polyketide synthase from *Clohesyomyces aquaticus* (Pleosporales) (71%), and a polyketide synthase of *Metarhizium acridum* (Sordariomycetes) (69%). PKS-NPRS 58882 had amino acid homology of 67–81% with a variety of fungi, and only the top three hits are within the order Pleosporales (Table 3, Figure 1).

**Figure 2:**
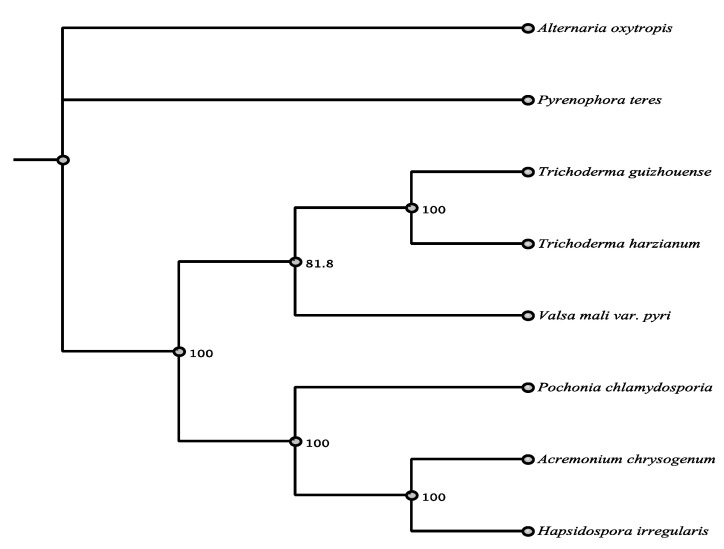
Maximum parsimony tree (MUSCLE alignment and 1000 replicates). *Trichoderma guizhouense* was used as the outgroup. Fungi in tree are pblast results of 21438 sequence.

PKS-NRPS 5601 was most similar to a hypothetical polyketide synthase of *Setosphaeria turcica* (Pleosporaceae, Pleosporales, Dothideomycetes) according to BLASTn and BLASTp (Figure S2). Alignments with the hypothetical mRNA and protein sequences resulted in identity matches of 67.1% and 69%, respectively. The second closest BLASTp match did not fall within the Pleosporales and the remainder did not fall within the Dothideomycetes. PKS-NRPS 21438 was most similar to a hypothetical protein from *Pyrenophora teres* (Pleosporaceae, Pleosporales, Dothideomycetes) by BLASTn and BLASTp (Figure S3). Alignment identities were 87% for nucleic acid and 88% for amino acid. The closest match to PKS-NRP 21438 with a known function by BLASTp was *Sch210972*, that inhibits cytokine receptors, from *Hapsidospora irregularis* (Sordariomycetes) with 52% amino acid identity. Only the closest BLASTp match, *Pyrenophora teres* is within the Pleosporales (Table 4) and all the other matches fell within the Sordariomycetes at or below 52% (Figure 2). PKS-NRPS 62407 was most similar by BLASTn to mRNA for hypothetical proteins from *Uncinocarpus reesii* (Eurotiomycetes) with 68.7% identity, and the top BLASTp hit was to a PKS-NRPS of *Tolypocladium ophioglossoides* (Sordariomycetes), with 64% identity (Figure S4).

### 3.3. Type I PKS

Thirteen *PKS-*like genes were of the fungal iterative Type I PKS as characterized by linear organization with different levels of reducing capabilities depending on the presence or absence of beta-keto domains. Of the 13, seven were identified as highly reducing, four as partially reducing, and two as non-reducing (Table 1). Some of the type I PKSs of *A. oxytropis* are potential orthologs to PKSs of known function from other fungi. The majority of the others were closely related to genera from the Ascomycota that are associated with plants, but with unknown function identified for the particular PKSs.

#### 3.3.1. Highly Reducing Type I PKSs

**Figure 3:**
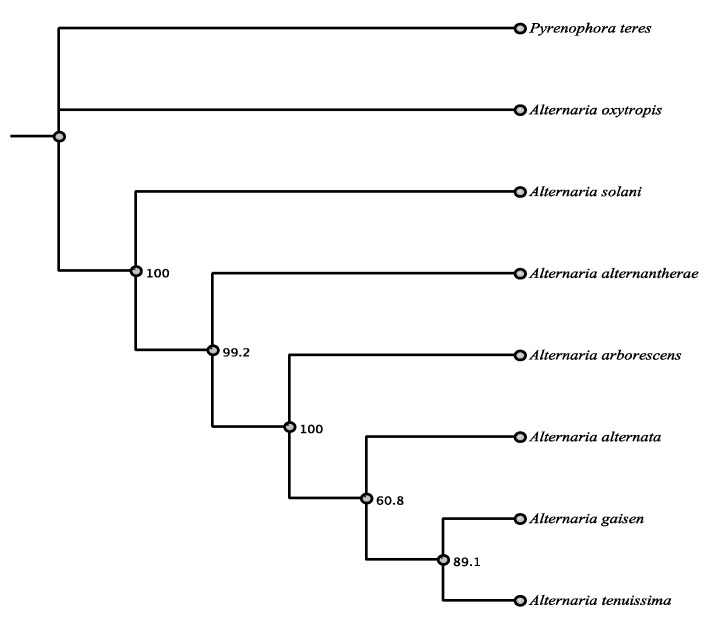
Maximum parsimony tree (MUSCLE alignment and 1000 replicates). *Alternaria arborescens* was used as the outgroup. Fungi in tree are pblast results of 2122 sequence.

Among the identified highly reducing type I PKSs, PKS 2122 had the highest identity with a protein of known function. A megablast search of the NCBI nucleotide database identified highest identity (83.8%) to the *alt5* gene of *Alternaria solani* (Pleosporaceae, Pleosporales) and 90% amino acid identity. PKS 2122 was identified in both AntiSMASH and the CDD to have the domains KS, AT, DH, MT, ER, KR, and ACP as a type I, HR-PKS which are the same domains that are present in *alt5* in the same order with a similar size of linker regions (Figure S5). The *alt5* gene encodes for PKSN, the enzyme responsible for producing the compound alterapyrone [30]. PKS 2122 was very highly conserved and had very high amino acid identity of 89–90% with the top seven matches that were all *Alternaria* spp. or relatives (Table S1, Figure 3).

PKS 1562 was most similar to *Bipolaris maydis* (Pleosporaceae, Pleosporales) by BLASTn to *Pks5*, a gene that encodes a PKS with no known function. PKS 1562 has the domains KS, AT, DH, MT and ER from the CDD. Alignment of PKS 1562 with PKS*5* gave 85% amino acid identity match. Comparing the amino acid sequences of both on the CDD showed that the KS, AT, and DH domains and the linking regions therein were highly similar whereas *Pks5* contains a different MT domain as well as a KR domain (Figure S6). PKS 1562 has only a partial KR domain at the 3′ end.

**Figure 4:**
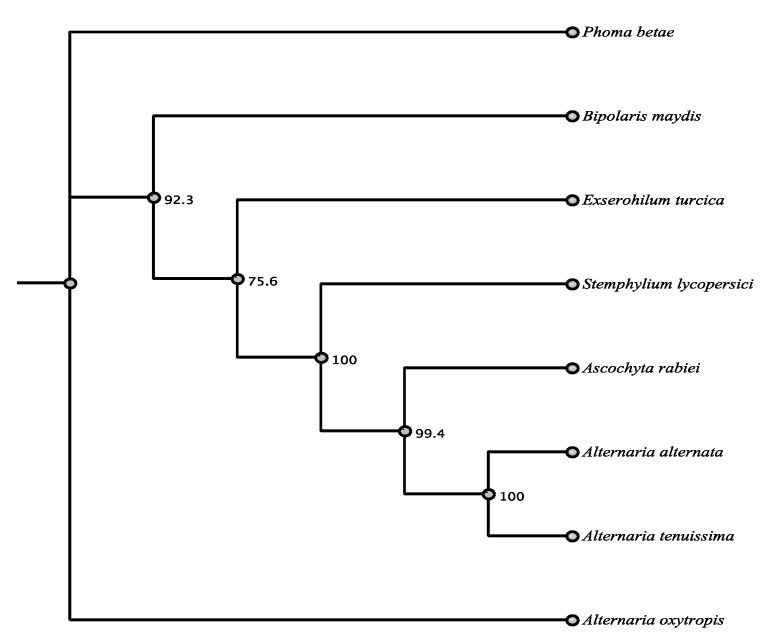
Maximum parsimony tree (MUSCLE alignment and 1000 replicates). *Bipolaris maydis* was used as the outgroup. Fungi in tree are pblast results of 96133 sequence.

Both highly reducing type I PKS 59499 and PKS96133 are similar to PKSs from other toxin producing species. PKS 59499 showed 67.7% identity in a BLASTn search with *Aspergillus nomius* (Eurotiomycetes) and an amino acid identity of 66%. PKS 96133 was most closely related by BLASTn to *pksF* of *Alternaria alternata* (Pleosporaceae, Pleosporales) another known toxin producer (Figure S7). Alignment of DNA and proteins found identity of 80.1% and 88% between PKSF and PKS 96133. The domains KS, AT, DH, and MT were the same as seen with CDD comparison though the order was different (Figure S8). The function of PKSF is not known, but PKS 96133 was highly conserved and had very high amino acid identity of 85–88% with the top seven matches, which were all *Alternaria* spp. or other Pleoporales (Table 5, Figure 4).

**Figure 5:**
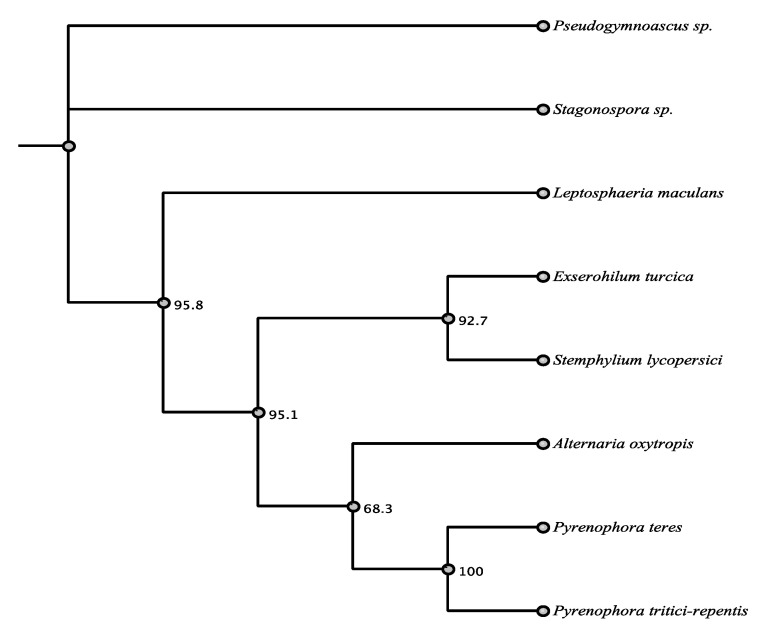
Maximum parsimony tree (MUSCLE alignment and 1000 replicates). *Stagonospora* sp. was used as the outgroup. Fungi in tree are pblast results of 39849 sequence.

The highly reducing PKS 39849 was most similar to phenolpthiocerol polyketide synthase *ppsA* of *Pyrenophora teres* (Pleosporaceae, Pleosporales) with 71.3% nucleotide identity and 74.5% amino acid identity (Figure S10). The closest BLASTp matches are within the Pleosporaceae and all fall within the Pleosporales (Table 6, Figure 5).

#### 3.3.2. Partially Reducing Type I PKS

The partially reducing PKS 17612 has 64% amino acid identity with a hypothetical protein from *Penicillium antarcticum* (Eurotiomycetes) and 63% identity with *Cladosporium cladosporioides* (Dothideomycetes) cla2 PPSB. CLA2 encodes the highly reducing polyketide synthase cladosporin biosynthesis cluster. None of the top seven BLASTp matches fell within the order Pleosporales and only one fell within the Dothideomycetes (Table S2, Figures S11 and S23).

**Figure 6:**
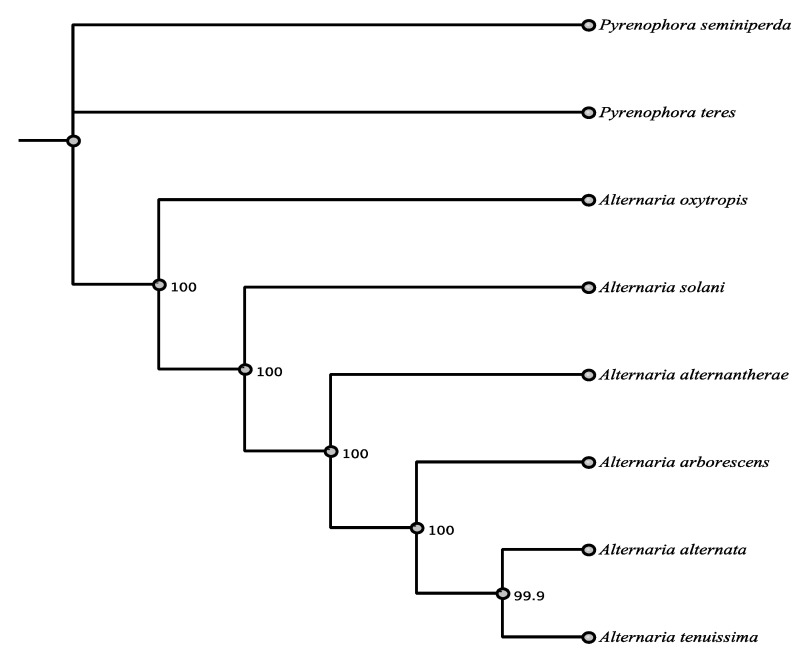
Maximum parsimony tree (MUSCLE alignment and 1000 replicates). *Pyrenophora seminiperda* was used as the outgroup. Fungi in tree are pblast results of 8407 sequence.

PKS 8407 has 80.2% nucleotide identity with the *pksF* polyketide synthase of *Alternaria solani* (Pleosporaceae, Pleosporales) by BLASTn. Amino acid identity with PKSF, a highly reducing PKS, was 81%. PKS 8407 contains the domains KS, AT, DH, ER, ACP and CDD match shows domains that are very similar (Figure S12). PKS 8407 had moderately conserved amino acid identity of 69.4–81.1% with the top seven matches that were all *Alternaria* spp. or relatives (Table S3, Figure 6).

**Figure 7:**
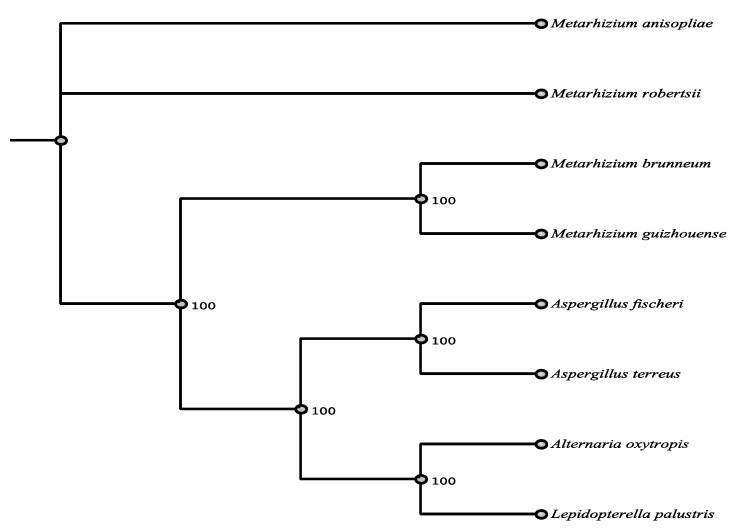
Maximum parsimony tree (MUSCLE alignment and 1000 replicates). *Aspergillus fischeri* was used as the outgroup. Fungi in tree are pblast results of 3398 sequence.

PKS 3398 was most closely related by BLASTn to a partial mRNA from *Aspergillus eucalypticola* (Eurotiomycetes) (66% identity), *Epichloe festucae* Fl1 (Sordariomycetes) (66% identity), *Metarhizium robertsii* beta-ketoacyl synthase (Sordariomycetes) (66% identity), and *Metarhizium brunneum* beta-ketoacyl synthase (68% identity, but more gaps). The highest protein identity was to *M. brunneum* with 64% amino acid identity (Figure S14). None of the matches fell within the order Pleosporales, and only one fell within the Dothideomycetes (Figure 7, Table S4).

#### 3.3.3. Non-Reducing Type I PKS

**Figure 8:**
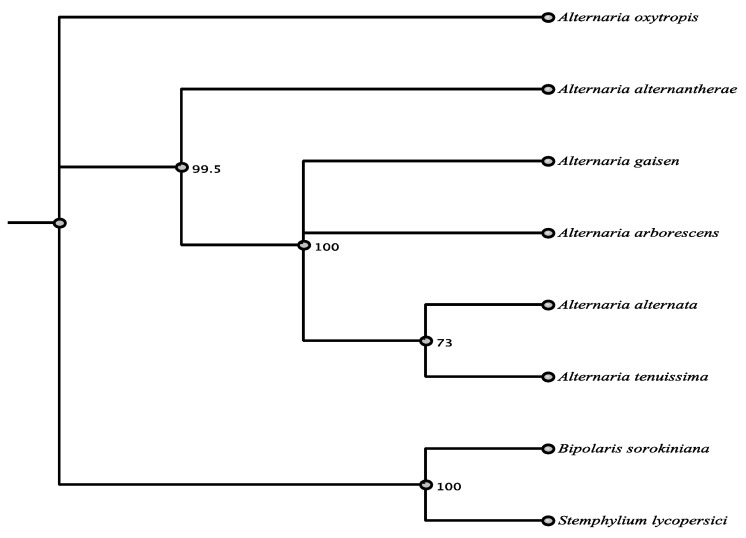
Maximum parsimony tree (MUSCLE alignment and 1000 replicates). *Stemphylium lycoperScheme 40283* sequence.

The non-reducing Type I PKS 40283 has high nucleotide (86.6%) and amino acid (95%) identity to pksA of *Alternaria alternata* (Pleosporaceae, Pleosporales). PKS 40283 has the domains KS, AT, two ACP, and TE and the domain order is identical by CDD (Figure S13). Prediction of secondary metabolite products indicated that PKS 40283 likely produced melanin (Table 2), thus PKS 40283 is likely responsible for melanin production in *A. oxytropis*. The identity match with PKSA of *Alternaria alternata*, along with other melanin producing PKS from fungi, leads to this function for PKS 40283. *PksA* in *A. alternata* functions as the biosynthetic pathway for melanin production [31,32]. PKS 40283 was very highly conserved had very high amino acid identity of 88.9–95.3% and the top five matches were *Alternaria* spp. (Table S5, Figure 8).

PKS 29103 was most closely related by BLASTn to a gene encoding a hypothetical protein of *Thielavia terrestris* (Sordariomycetes) with 65.9% identity (Figure S22). A BLASTp search yielded a hypothetical protein from *Epicoccum nigrum* (Pleosporaceae, Pleosporales) with 70% amino acid identity and *pks27*, which codes for Asparasone A from *Aspergillus flavus* (Eurotiomycetes) with 61% identity.

### 3.4. Non-Ribosomal Peptide Synthases

**Figure 9:**
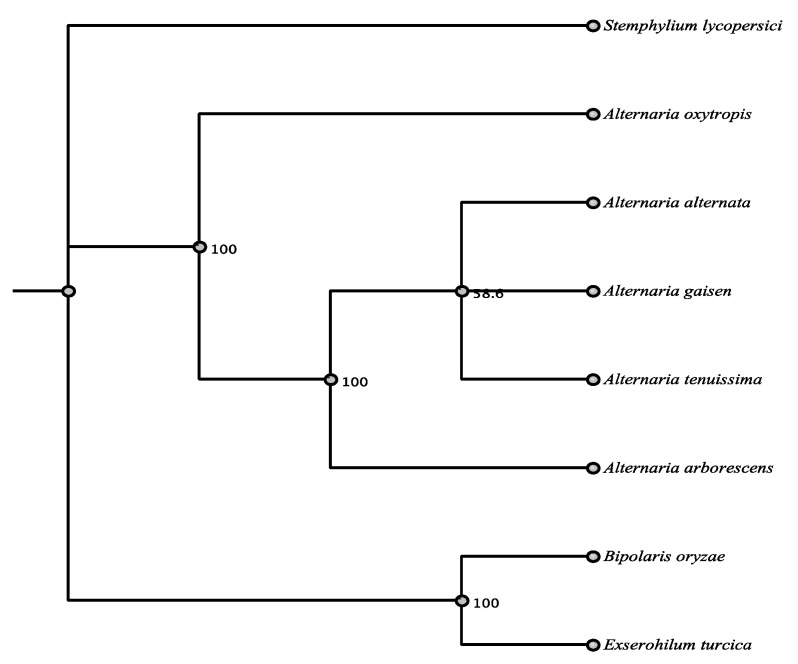
Maximum parsimony tree (MUSCLE alignment and 1000 replicates). *Bipolaris oryzae* was used as the outgroup. Fungi in tree are pblast results of 33635 sequence.

Of the five non-ribosomal peptide synthases found in the *A. oxytropis* genome, the function of NRPS 33635 is best understood because of its 84.6% nucleotide identity with the NPS6 gene of *Alternaria alternata* (Pleosporaceae, Pleosporales) and 91% amino acid identity with *Alternaria alternata* NRPS by BLASTp. NRPS 33635 has the domains A, PCP, C, two PCPs, and C with both CDD and antiSMASH. Comparison with CDD shows that the proteins are almost identical (Figure S15). NRPS 33635 was highly conserved and had high amino acid identity of 82.6–91.5% and the top 4 matches that were *Alternaria* spp. and the next three matches were Pleosporales (Table S6, Figure 9). Contig 33635 (Table 2, Figure S15), was predicted to cyclize. It has an adenylation domain (A), peptidyl carrier protein (PCP, or PP-in the figures), and ends with a condensation-like domain (plain condensation domain in figures). This agrees with the predicted product of dimethyl coprogen, a siderophore produced by *A. longipes* [33].

NRPS 5682 had 85% identity with *Alternaria brassicae nrps1* gene by BLASTn. BLASTp gave the highest matches with the NRPS1 of *A. alternata* with 91% amino acid identity and *A. brassicae* with 89% identity. NRPS 5682 represents a partial sequence and so contains fewer domains than are found for *A. brassicae* NRPS1 (Figure S16). Domains in the following order: A, PCP, C, A, PCP, E, C, and A were identified for NRPS 5682. NRPS5682 was highly conserved and had high amino acid identity of 79.2–90.8% with the top seven matches, which were all *Alternaria* spp. or relatives (Table S7, Figure S24). NRPS 8194 showed the highest nucleotide match from BLASTn and BLASTp searches to an mRNA of *Pyrenophora tritici repentis* (Pleosporaceae, Pleosporales) HC-toxin synthetase with 85% nucleotide identity and 87% amino acid identity. NRPS 8194 has the domains PCP, C, A, PCP, C, and A with both CDD and antiSMASH. Comparison on CDD of HC-toxin synthetase with NRPS8194 shows that the domains are similar but NRPS 8194 could be a truncated form of HC-toxin synthetase (Figure S18). All protein matches fell within the Pleosporaceae. Contigs 5682 (Figure S16) and 8194 (Figure S18), are partial contigs that are closely related to AAP78735 and XP_001939433, respectively. These close matches have an A domain, C-domain, PP domain, and end with a C-like domain. NRPS 5682 is closest match to HC toxin synthetase, a cyclic tetrapeptide, which agrees with the predicted cyclization. In contrast, little is known about the structure of the *nrps*1 gene of *A. brassicae*, which is the closest match to NRPS 8194.

NRPS 7859 had 82.7% nucleotide identity and 76% amino acid identity with the *nrps9* gene from *Bipolaris maydis* (Pleosporaceae, Pleosporales). NRPS 7859 contains the domains A, PCP, C, and A with both CDD and antiSMASH and comparison with CDD also showed similarity of domain order (Figure S17). The top four protein matches fell within the Pleosporaceae and the rest within the Eurotiomycetes. NRPS 40703 showed highest BLASTn and BLASTp matches with NRPS2 of *Alternaria alternata* (Pleosporaceae, Pleosporales) with 82% nucleotide identity and 82% amino acid identity. Contig 40703 (Figure S19), lacks a PP (PCP) domain, so is not predicted to cyclize and the final product of NRPS 40703 has not been identified.

### 3.5. A Domain Substrates

The adenylation domain is a catalytic domain that is important for the biosynthesis of natural product peptides. The substrate engages first with the A domain before it is incorporated with the peptide natural product [34]. Every A domain selects a unique substrate [24,26]. Prediction of the A domain substrates (Table 2) showed that almost every A domain had at least one Phe. Two contigs were exceptions; for contigs 58,882 and 62407, the predicted substrates were Gly and Tyr, respectively. Some contigs had multiple A domains, each with its predicted substrate. Contig 5682 was predicted to contain three A domains, each with a Phe substrate, contig 7859 also had three A domains with substrates Pro, Try, Try (in that order), and contig 40703 had four A domains with substrates aminoadipate, Phe, Phe, Val, (in that order). Contig 33635 (Table 2), had Phe as a predicted substrate which is consistent with the predicted product of dimethyl coprogen. Contig 8194 was predicted to contain three A domains with substrates Try, Pro, Phe, however the closest match, HC toxin synthase has 4 domains and one uses Pro as a substrate and two use Ala. Since a final SM product has not been determined for NRPS 5682, 7859 and 40703, prediction of their A substrates cannot be verified.

### 3.6. Phylogenetic Relationships

Analysis of the closest relationships for each SM tested revealed surprising levels of diversity. *Alternaria* falls within the family Pleosporaceae, the order Pleosporales, and the class Dothideomycetes; 68% of the secondary metabolites gave a closest top match with the same genus, family, order, or class as *A. oxytropis*. Phylogenetic relationships among closest amino acid matches with *Alternaria* sp. were for seven highly conserved (81–95% aa identity) PKS and NRPS: PKS 2122, PKS 96133, PKS 8407, PKS 40283, NRPS 33635, NRPS 5682, and NRPS 40703. These PKS and NRPS, marked as “A” in Table 1, are all well characterized with known functions and clear inheritance lines. Five SM were most closely related to other members within the Pleosporaceae family; PKS-NRPS 58882, PKS 1562, PKS 39849, NRPS 7859, and NRPS 8194. These were marked as “P” in Table 1 and showed intermediate to high conservation (76–87% aa identity) with the top match. Three PKS and PKS-NRPS, PKS-NRPS 5601, PKS-NRPS 21438, and PKS 29103, were most closely related to a single member of the Pleosporales order with the remaining matches to fungi that did not fall within the Dothideomycetes. These were marked as “M” in Table 1 and showed intermediate to high conservation (69–88% aa identity) with the top match. Seven PKS and PKS-NRPS showed no identity with any members of the Pleosporales or the class Dothideomycetes; PKS-NRPS 62407, PKS 59499, PKS 9132 (Table S8, Figures S9 and S25), PKS 12778 (Figure S20), PKS 3398, and PKS 42460 (Figure S21) showed low to intermediate conservation (64–73% aa identity) with their closest match.

## 4. Discussion

The number of PKSs identified in this work for *A. oxytropis*, 22, is lower compared to some toxin-producing fungi. *Aspergillus* species have been identified with high numbers of PKS and NRPS gene clusters (33–81) [8,10,16] and *Fusarium* species with 26–32 gene clusters [15]. Lu et al. [35] found motifs for 59 possible PKSs for *A. oxytropis*, but did not further confirm or identify them, and thus the higher number could be due to pseudogenes.

The presence of KSD and CD (Table 2) correlated with the presence of PKS, and condensation domains as shown in the figures. The contigs (Supplemental Figures and Table 2, show the presence of a KS domain. Contigs from Figures S2–S4 and S15–S20, and Table 2 contain a condensation domain. Both C and KS domains are highly conserved. Type I PKS, modular and hybrid, are described by C and KS domain phylogeny [25].

Cyclization of final peptide product requires the presence of a condensation (C), adenylation (A), and end with a thiolation (T) domains. Fungi NRPS lack a terminal thioester (TE) domain. Instead, macrocyclic fungal NRPS end with a condensation-like domain (Ct), which corresponds, in function, to TE [36]. Requirements for cyclization include the presence of a C domain, A domain, T domain (PCP, peptidyl carrier protein), and a terminal Ct domain (with or without an attached PP arm) [36,37,38,39]. Cyclization predictions were confirmed for the NRPS in which a product had been identified. NRPS 33635 satisfied all requirements for cyclization and the product is cyclized. NRPS 8194 is a partial sequence, and its closest matches fulfills the cyclization requirements in producing a cyclic tetrapeptide. Similarly, NRPS 5682 is a partial sequence, and its closest match fulfills the cyclization requirements, however the identity of the product has not been confirmed. Though some other contigs included all three domains required for cyclization, they did not end with a Ct domain, or lacked a PCP domain. NRPS 40703 (Figure S19), lacks a PP (PCP) domain, but has an ACP-like domain. While ACP domains (acyl-carrier protein) resemble the T domain of NRPS [40] it is not required for cyclization, which suggests that this contig would not enzymatically cyclize its end product.

The A domain of NRPS are required for the biosynthesis of the peptide natural product, and substrate selection is determined by the A domain. All basic NRPS must have an A domain, required for the biosynthesis of the peptide natural product, and substrate selection; a PCP (thiolation) domain, transports and attaches the substrates to different catalytic domains; and a C domain, that catalyzes peptide bond formation [34,40]. While amino acids substrates were predicted, since few final products have been identified, it was difficult to determine the accuracy of some of the predictions.

Seven of the 22 PKSs identified were most similar to an *Alternaria* species. This was not unexpected as *A. oxytropis* is most closely related to other *Alternaria* species and *Alternaria oxytropis* falls within the Pleosporaceae. Among the seven PKSs most similar to other *Alternaria* species, they were distributed among the highly reducing PKS, partially reducing PKS, nonreducing PKS, and NRPS. Other fungi with closest matches within the Pleosporaceae were *Pyrenophora*, *Bipolaris*, and *Setsosphaeria*. There were four PKS from the genus *Pyrenophora*. *Bipolaris* spp. provided the closest match for two PKS. *Fusarium* with two matches was the fungus outside of the Pleosporaceae with the highest number of matches.

Several of the PKS/NRPS identified (PKS 2122, PKS 8407, NRPS 5682) showed high identity with *Alternaria* species or relatives through the first seven matches and three others, PKS 40283, PKS 96133, and NRPS 33635) showed high identity with *Alternaria* species and other Pleosporales. These PKS and NRPS are obviously highly conserved among *Alternaria* sp. and Pleosporales. For several of the sequences, including PKS 8407, NRPS 3365, *A. oxytropis* clustered with other *Alternaria* spp. in the phylogenetic trees. In other phylogenetic trees (PKS 40283, PKS 96133) *A. oxytropis* did not cluster with other *Alternaria* spp.

For *A. oxytropis*, 68% of the SM genes did not match the genus *Alternaria* even at the amino acid level. Four sequences (PKS-NRPS 58882, PKS-NRPS 21438, NRPS 8194, and PKS 39849) had moderately high identity with *Pyrenophora* species in the family Pleosporaceae, and the rest of the matched fungi did not fall within the order Pleosporales. Two of those (PKS-NRPS 21438 and NRPS 8194) showed high conservation with 87–88% aa identity with *Pyrenophora* spp. Most *Pyrenophora* are pathogenic on cereals and the genus is monophyletic [41]. *Pyrenophora* is a sexual state of what was previously known as *Drechslera.* Even for sequences in which there is high identity with *Alternaria* species, such as PKS 2122 and NRPS 5682, the phylogenetic trees suggest that *A. oxytropis* is as closely related to *Pyrenophora* as to *Alternaria* spp. This raises questions about the inheritance of these SM and the taxonomic placement of the fungus within the genus *Alternaria*. This high sequence conservation suggests a potential taxonomic lineage for *Alternaria oxytropis*, perhaps as a product of hybridization or recombination between *Alternaria* and *Pyrenophora* ancestors.

Three sequences analyzed, PKS 9132, PKS 17612, and PKS 3398, had no closest matches in the Pleosporales. For the PKS 9132 sequence, the phylogenetic trees show no clustering of *A. oxytropis* with any of the closest matches, whereas for the other two sequences, *A. oxytropis* clustered with otherwise unrelated fungi.

Within the phylum Ascomycota, the large majority of SM gene clusters are found only in closely related species [8]. Those few that are more broadly distributed across the phylum are often highly divergent between even closely related species [22]. Rokas et al. [42] suggested several possible explanations for SM cluster variability based on molecular evolutionary processes including functional diversity, horizontal gene transfer, and de novo assembly. For *A. oxytropis* SM, functional diversity is likely, horizontal gene transfer is possible, and no evidence was found for de novo assembly.

Orthologous or paralagous functional diversity could explain some of the variability found in *A. oxytropis* SM, particularly for those with the closest matches to members of the Pleosporaceae, but not *Alternaria*, marked as P in Table 1. All five of these SM genes showed slight divergence in domain size or order from their closest matches. NRPS 8194 differs slightly in gene order from that of the HC-toxin synthetase gene of *Pyrenophora tritici-repentis* and *A. oxytropis* is not known to produce HC toxin. The SWN gene cluster of swainsonine-producing fungi vary in the order of genes other than *swnK* (PKS-NRPS 58882) in the cluster [6].

The *A. oxytropi*s SM genes that did not match fungi within the order Pleosporales or did not match fungi within the class Dotheomycetes have a more speculative ontogeny. Some of the variability could be to due to horizontal gene transfer or more likely a combination of horizontal gene transfer and orthologous or paralagous changes. The domains (and order of domains) in these *A. oxytropis* SM genes are similar to their closest matching fungi. Horizontal gene transfer between fungal classes has been speculated for the entire sterigmatocystin biosynthetic pathway, that appears to have been transferred from *Aspergillus nidulans* to *Podospora* [43]. A study of SM within *Aspergillus fumigatus* showed that 13 of the SM gene clusters were generally conserved with low variation and 23 were highly variable [8]. Those authors found six examples of gene content polymorphisms that were exemplified by loss of gene cluster function, structural changes in the metabolite, or change in the expression or transport of the metabolite.

Manning et al. [44] reported that the genome of *Pyrenophora tritici-repentis* includes several NRPSs that may have been derived by horizontal gene transfer and gene duplication. The polyketide host-selective toxins associated with *Alternaria* spp. adapted to pear, apple, tangerine, citrus, rough lemon, and tomato are all found on conditionally dispensable chromosomes (CDC) that could have been acquired through horizontal chromosome transfer among the *Alternaria* spp. [45]. Armitage et al. [46] showed that while *A. tenuissima* pathotypes shared 10 types of transposable elements (TE) with *A. arborescens*, the pathogens contained significantly decreased numbers of TE in the DDE and gypsy families, and significantly higher numbers of TE in the mariner family. They speculated that the TEs may have increased the variability in the fungi. Determining if gain of whole gene clusters through TE or horizontal chromosome transfer might be possible explanations for SM sequences in *A. oxytropis* would require many more sequences from related species, chromosome mapping of the fungus, analysis of the number and location of TE, and functional analyses of the SM.

The closest matches for the *A. oxytropis* PKSs were for *Alternaria* species for highly conserved genes. PKS40283 had the highest amino acid identity (95%) of any of the PKSs identified, with *A. alternata* PKSA, which is responsible for melanin production. The gene responsible for melanin production is highly conserved among many dark-colored fungi. Although *A. oxytropis* is dark black in culture and *A. cinerea* and *A. fulva* are tan and grey, respectively, in culture [29], the latter two fungi have the same sequence as identified for PKS40283. Silencing of the homologous gene, *pks1*, in *Slafractonia leguminicola*, also within the family Plesoporaceae and a swainsonine toxin producer, caused a reduction in melanin synthesis and relevant transcript levels [47].

NRPS 3365 also had high amino acid homology (91%) with *Alternaria alternata* NPS6. This is likely a highly conserved NRPS among both pathogenic and saprobic fungi, including pathogenic and nonpathogenic isolates of *Pyrenophora tritici-repentis* [44]. NPS6 of *Cochliobolus* spp. was found to have orthologs in all filamentous fungi examined [48]. It was also found to affect virulence since loss of expression reduced virulence but did not completely abolish it. Additionally, lack of expression led to an increase in sensitivity to hydrogen peroxide. In *Alternaria alternata*, *nps6* is necessary for the biosynthesis of dimethyl copreogen siderophores as well as functioning as a virulence factor [49]. This was the predicted SM for *A. oxytropis* for NRPS 3365 as well. Multiple transcription factors control *nps6* transcript accumulation in *A. alternata* including NADPH oxidase, a redox responsive transcription facto YAP1 and a mitogen activated protein kinase HOG1 [50]. Mutation of *nps6* or any of the transcription factors results in increased sensitivity to reactive oxygen species (ROS) and reduced virulence in citrus. ROS sensitivity can be partially rescued though with the addition of iron. The effects of iron and ROS sensitivity lead to the idea that NPS6 is important to the production of siderophores involved in iron uptake [50]. Deletion of NPS6 in *Fusarium graminearum*, *Cochliobolus miyabeanus*, and *A. brassicicola* also resulted in the same responses of increased sensitivity to ROS and reduced virulence [51].

NRPS 5682 also showed the same high level of amino acid identity (91%) with *Alternaria alternata* NRPS1. *Alternaria* NRPS1 is likely involved in plant infection since increased expression of *nrps1* was found during host infection by *A. brassicae* [52]. This NRPS could play some role in the establishment of the endophytic relationship of *A. oxytropis* in its plant host. PKS2122 showed a high level of amino acid identity (90%) with *Alternaria solani* PKSN. PKSN is the product of the *alt5* gene, which is essential for alterapyrone biosynthesis [30]; when *alt5* was expressed in *Aspergillus oryzae* under an alpha-amylase promoter, alternapyrone was produced. Because there was a high level of identity at both the nucleotide and protein levels between PKS2122 and PKSN, it is likely that *A*. *oxytropis* also produces this compound. PKS2122 was very highly conserved among many *Alternaria* species. PKS-NRPS 5882 was verified as the *swnK* KS [6]. Two of the fungi with high amino acid identity, *Pyrenophora seminiperda* and *Clohesyomyces aquaticus* have not been reported to produce swainsonine or be associated with mammalian toxicity.

Both the number of PKS, along with the lack of associated functions identified here, are common to studies in other fungi. While additional PKS genes or PKS-like genes might be identified from the completed genome sequence of *A. oxytropis*, it is likely that the majority of the PKS genes have been identified here. Interestingly, the functions of the 22 genes found in this study remain unknown. Although secondary metabolites have been studied in many fungi, and the genomes of many fungi have been sequenced, it is significant that functions for these have not been readily identified. Transcriptomic and metabolomic studies will likely be necessary to better understand the function of the genes identified in this work.

## 5. Conclusions

Secondary metabolite production is essential to most fungi, and especially so to those fungi that produce toxins. The PKS, NRPS, and PKS/NRPS identified for *A. oxtryopis* show the conservation of these genes within the genus *Alternaria* and order Pleosporales. The PKS gene for melanin exemplifies very high conservation. The data presented here also highlight the lack of information on how these genes are derived, spread, and diversified. The PKS and PKS/NRPS identified here that have no close matches within the Dothideomycetes are examples that need additional work to help explain their ontogeny. Unless a fungus is economically important, it is unlikely to have been sequenced. Sequencing of many other fungi within the Dothideomycetes will help to identify some conservation between the poorly characterized secondary metabolite genes and more highly characterized PKS, NRPS, and PKS/NRPS, and thus closing the knowledge gap.

## Figures and Tables

**Table 1:** Polyketide synthases and non-ribosomal peptide synthases of *Alternaria* section *Undifilum oxytropis* with highest Blastp hits and % identity match of amino acid sequences.

Type	Contig	Genbank Accession	% Identity	BLASTp Hit	Accession	Protein Match Category
PKS-NRPS	58882	KY365741	81	*Slafractonia leguminicola*	AQV04236	M
hybrid	5601	MK058350	69	*Setosphaeria turcica*	XP_008025924	M
	21438	MK058352	88	*Pyrenophora teres*	XP_003303559	M
	62407	MK058351	64	*Tolypocladium ophioglossoides*	KND87119	N
Type I, highly	2122	MK471354	90	*Alternaria solani*	Q5KTM9	A
reducing	1562	MK495800	85	*Bipolaris maydis*	AAR90260	P
	59499	MK492487	66	*Aspergillus nomius*	KNG90368	N
	96133	MK492486	88	*Alternaria alternata*	AFN68297	A
	9132	MK450599	73	*Fusraium aywerte*	ALQ32761	N
	39849	MK492485	76	*Pyrenophora tritici-repentis*	PZC93669	M
	12778	MK471353	65	*Fusarium oxysporum f. sp. conglutinans*	EXL67078	N
Type I, partially	17612	MK495801	63	*Cladosporium cladosporioides*	AOAOYOM151	N
reducing	8407	MK492484	81	*Alternaria solani*	Q2ABP6	A
	3398	MK492483	64	*Metarhizium brunneum*	KID62944	N
	42460	MK507973	46	*Colletotrichum fructicola*	ELA32259	N
Type I,	40283	MK402071	95	*Alternaria alternata*	AFN68292	A
non-reducing	29103	MK471352	70	*Epicoccum nigrum*	OSS52043	M
NRPS	33635	MK409655	91	*Alternaria alternata*	AFN69082	A
	5682	MK468800	91	*Alternaria alternata*	XP_0188382376	A
	7859	MK426697	76	*Bipolaris maydis*	AAX09991	P
	8194	MK468799	87	*Pyrenophora tritici-repentis*	XP_001939433	P
	40703	MK471351	82	*Alternaria alternata*	XP_018389223	A

% query cover (percentage of the protein that was included in the match) averaged 99.1% (range 97–100); A = *Alternaria* genus 1–4 >Pleosporacae family; P = Pleosporaceae family 1–4; M = Pleosporales order 1–2 > not Dothidiomycetes class; N = not Pleosporales order/not Dothidiomycetes class.

**Table 2:** SeMPI, antiSMASH, NaPDoS, NRPSep, and IntrProScan predictions for *Alternaria oxytropis* PKS and NRPS domains. The predictions show the presence or absence of condensation domain (CD), and ketosynthase domain (KSD); the predicted substrates for the adenylation domain; and the enzymatic cyclization of the final product.

Contig	Genbank Accession	CD	KSD	Adenylation Domain Substrate	Cyclization
58882	KY365741	−	+	Gly	No
5601	MK058350	+	+	Phe	No
21438	MK058352	+	+	Phe	No
62407	MK058351	+	+	Tyr	No
12778	MK471353	+	+	Phe	No
33635	MK409655	+	−	Phe	Cyclize
5682	MK468800	+	−	Pro, Pro, Phe	Likely
7859	MK426697	+	−	Pro, Try, Try	No
8194	MK468799	+	−	Try, Pro, Phe	Likely
40703	MK471351	+	−	Aminoadipate, Phe, Phe, Val	No

**Table 3:** Percent amino acid (aa) identity of pblast results of PKS-NRPS 58882 sequence. Fungal order within the class Dothideomycetes (D) or other fungal class as listed.

Organism	Accession	Order/Class	% aa Identity
*Pyrenophora seminiperda*	RMZ73569.1	Pleosporales/D	80.9%
*Slafractonia leguminicola*	AQV04236.1	Pleosporales/D	72.7%
*Clohesyomyces aquaticus*	ORY11783.1	Pleosporales/D	71.0%
*Metarhizium acridum*	XP_007815889.1	Sordariomycetes	69.0%
Chaetothyriaceae	AQV04224.1	Eurotiomycetes	69.1%
*Pseudogymnoascus* sp.	KFY51099.1	unknown/D	67.3%
*Rosellinia necatrix*	GAP93000.1	Sordariomycetes	67.0%

**Table 4:** Percent amino acid identity of pblast results of PKS-NRPS 21438 sequence. Fungal order if within the class Dothideomycetes (D) or other fungal class as listed.

Organism	Accession	Order/Class	% aa Identity
*Pyrenophora teres*	EFQ87243.1	Pleosporales/D	87.5%
*Acremonium chrysogenum*	KFH40930.1	Sordariomycetes	52.7%
*Hapsidospora irregularis*	AKG54858.1	Sordariomycetes	52.2%
*Pochonia chlyamydosporia*	XP_018142849.1	Sordariomycetes	69.2%
*Valsa mali* var. *pyri*	KUI56379.1	Sordariomycetes	52.0%
*Trichoderma guizhouense*	OPB40675.1	Sordariomycetes	48.2%
*Trichoderma harzianum*	PKK52345.1	Sordariomycetes	48.0%

**Table 5:** Percent amino acid (aa) identity of pblast results of PKS 96133 sequence. Fungal order within the class Dothideomycetes.

Organism	Accession	Order	% aa Identity
*Alternaria alternata*	AFN68297.1	Pleosporales	87.7%
*Stemphylium lycopersici*	RAR01042.1	Pleosporales	85.4%
*Alternaria tenuissima*	RYN28010.1	Pleosporales	86.9%
*Ascochyta rabiei*	KZM21157.1	Pleosporales	86.6%
*Phoma betae*	BAQ25466.1	Pleosporales	86.1%
*Bipolaris maydis*	AAR90270.1	Pleosporales	85.9%
*Exserohilum turcica*	XP_008031663.1	Pleosporales	87.1%

**Table 6:** Percent amino acid (aa) identity of pblast results of PKS 39849 sequence. Fungal order within the class Dothideomycetes.

Organism	Accession	Order	% aa Identity
*Pyrenophora teres*	EFQ87243.1	Pleosporales	74.5%
*Stemphylium lycopersici*	KNG47890.1	Pleosporales	73.9%
*Pyrenophora tritici-repentis*	XP_001937136.1	Pleosporales	72.9%
*Exserohilum turcica*	XP_008026829.1	Pleosporales	69.2%
*Leptosphaeria maculans*	XP_003842471.1	Pleosporales	66.8%
*Stagonospora* sp.	OAK96687.1	Pleosporales	60.4%
*Pseudogymnoascus* sp.	KFY81215.1	Unknown	50.3%

## Data Availability

Sequence data has been deposited into Genbank. See Table 1 for specific accession numbers.

## Supplementary Materials

The following are available online at https://www.mdpi.com/article/10.3390/jof7070538/s1, Figure S1: Domain order of PKS-NRPS 58882 and polyketide synthase of *Metarhizium acridum* XP_07815889, Figure S2: Domain order of PKS-NRPS 5601 and hypothetical protein of *Setosphaeria turcica* XP_008025924, Figure S3: Domain order of PKS-NRPS 21438 and hypothetical protein of *Pyrenophora teres* XP_003303559, Figure S4: Domain order of PKS-NRPS 62407 and hypothetical protein of *Tolypocladium ophioglossoides* KND87119, Figure S5: Domain order of PKS 2122 and ALT5 of *Alternaria solani* BAD83684, Figure S6: Domain order of PKS 1562 and PKS5 of *Bipolaris maydis* AAR90268, Figure S7: Domain order PKS 59499 and hypothetical protein of *Aspergillus nomius* KNG90368, Figure S8: Domain order of PKS 96133 and PKSF of *Alternaria alternata* AFN68297, Figure S9: Domain order of PKS 9132 and PKSD of *Aspergillus niger* XP_001394543, Figure S10: Domain order PKS 39849 and PPSA of *Pyrenophora tritici-repentis* XP_001937136, Figure S11: Domain order of PKS 17612 and PPSB of *Pyrenothora tritici-repentis* XP_001934720, Figure S12: Domain order of PKS 8407 and *Alternaria solani* PKSF BAE80697, Figure S13: Domain order of PKS 40283 and PKSA of *Alternaria alternata* AFN68292, Figure S14: Domain order of PKS 3398 and polyketide synthase of *Metarhizium brunneum* KID62944, Figure S15: Domain order of NRPS 33635 and NPS6 of *Alternaria alternata* AFN69082, Figure S16: Domain order of PKS 5682 and *Alternaria brassicae* nrps1 AAP78735, Figure S17: Domain order of NRPS 7859 and NPS9 of *Cochliobolus heterostrophus* AAX09991, Figure S18: Domain order of NRPS 8194 and HC-toxin synthetase of *Pyrenophora tritici-repentis*, Figure S19: Domain order of NRPS 40703 and NPS2 of *Alternaria brassicicola*, Figure S20: Domain order of PKS 12778 and hypothetical protein from *Fusarium oxysporum f. sp. conglutinans* EXL67078, Figure S21: Domain order of PKS 42460 and hypothetical polyketide synthase of *Colletotrichum gloeosporioides* XP_007270670, Figure S22: Domain order of PKS 29103 and hypothetical protein of *Aspergillus parasiticus* KJK63007, Figure S23: Maximum parsimony tree (MUSCLE alignment and 1000 replicates). *Lasiodiplodia theobromae* was used as the outgroup. Fungi in tree are pblast results of 17612 sequence; Figure S24: Maximum parsimony tree (MUSCLE alignment and 1000 replicates). *Pyrenophora teres was used as the outgroup. Fungi in tree are pblast results of 5682 sequence.:* Figure S25: Maximum parsimony tree (MUSCLE alignment and 1000 replicates). *Aspergillus luchuensis was used as the outgroup. Fungi in tree are pblast results of 9132 sequence.* Table S1: GenBank accession numbers and amino acid percent identity of blast results of 2122 sequence, Table S2: Genbank accession numbers and amino acid percent identity of blast results of 17612 sequence, Table S3: Genbank accession numbers and amino acid percent identity of blast results of 8407 sequence, Table S4: Genbank accession numbers and amino acid percent identity of blast results of 3398 sequence, Table S5: GenBank accession numbers and amino acid percent identity of blast results of 40283 sequence, Table S6: Genbank accession numbers and amino acid percent identity of blast results of 33635 sequence, Table S7: Genbank accession numbers and amino acid percent identity of blast results of 5682 sequence, Table S8: Genbank accession numbers and amino acid percent identity of blast results of 9132 sequence.
