# Supplementary material for: Genetic Relationships in the Toxin-Producing Fungal Endophyte, Alternaria oxytropis Using Polyketide Synthase and Non-Ribosomal Peptide Synthase Genes
Source: J Fungi (Basel). 2021 Jul 6;7(7):538. doi: 10.3390/jof7070538 (PMC8306250; doi:10.3390/jof7070538)
Supplement: Supplementary file 1 [file jof-07-00538-s001.zip › jof-1271709-supplementary.pdf]

Supplemental Figures

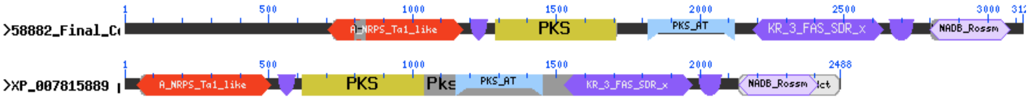

Figure S1. Domain order of PKS-NRPS58882 and polyketide synthase of *Metarhizium acridum* XP_07815889. A=adenylation, PKS = ketoacyl synthase, AT= acyl transferase, KR = ketoreductase, NADB Rossm = NAD binding Rossman folding.

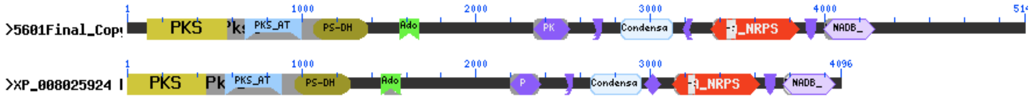

Figure S2. Domain order of PKS-NRPS5601 and hypothetical protein of *Setosphaeria turcica* XP_008025924. PKS = ketoacyl synthase, AT= acyl transferase, DH = dehydratase, Ado = methyltransferase, Condensa = condensation, A = adenylation, NADB = NAD binding.

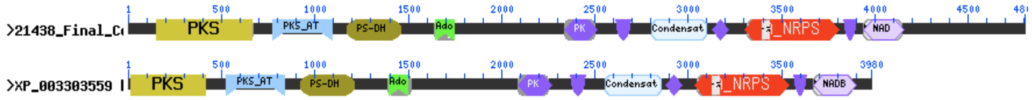

Figure S3. Domain order of PKS-NRPS21438 and hypothetical protein of *Pyrenophora teres* XP_003303559.

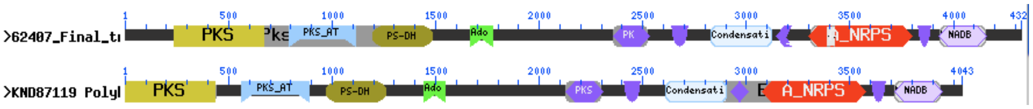

Figure S4. Domain order of PKS-NRPS62407 and hypothetical protein of *Tolypocladium ophioglossoides* KND87119.

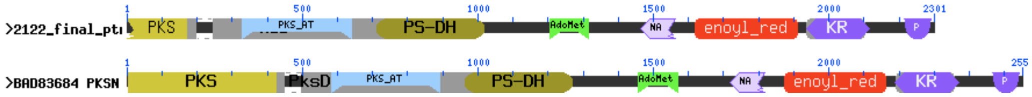

Figure S5. Domain order of PKS2122 and ALT5 of *Alternaria solani* BAD83684. PKS = ketoacyl synthase, AT= acyl transferase, DH = dehydratase, AdoMet = methyltransferase, NA = neuraminidase, enoyl red = enoyl reductase, KR = ketoreductase, P = acyl carrier protein.

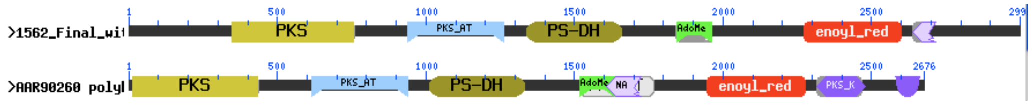

Figure S6. Domain order of PKS1562 and PKS5 of *Bipolaris maydis* AAR90268.

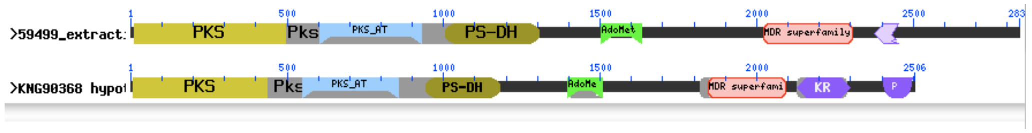

Figure S7. Domain order PKS59499 and hypothetical protein of *Aspergillus nomius* KNG90368. PKS = ketoacyl synthase, AT= acyl transferase, DH = dehydratase, AdoMet = methyltransferase, MDR superfamily = medium chain dehydrogenases/reductases, KR = ketoreductase, P = acyl carrier protein?

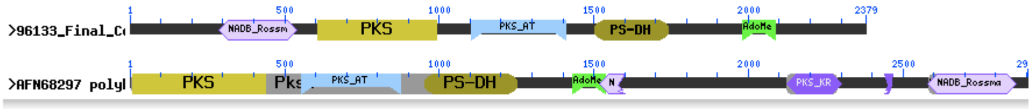

Figure S8. Domain order of PKS96133 and PKSF of *Alternaria alternata* AFN68297. NADB Rossm = NAD binding Rossman folding.

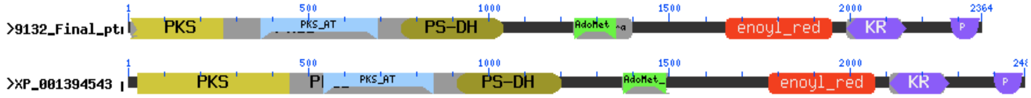

Figure S9. Domain order of PKS9132 and PKSD of *Aspergillus niger* XP_001394543. enoyl red = enoyl reductase, KR = ketoreductase, P = acyl carrier protein.

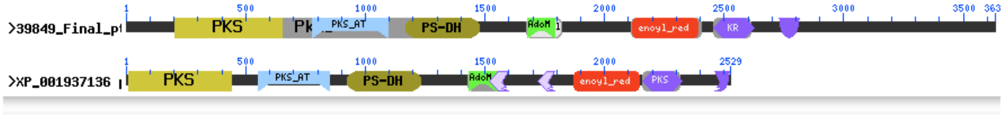

Figure S10. Domain order PKS39849 and PPSA of *Pyrenophora tritici-repentis* XP_001937136. PKS = keTable 11. Domain order of PKS17612 and PPSB of *Pyrenothora tritici-repentis* XP_001934720.

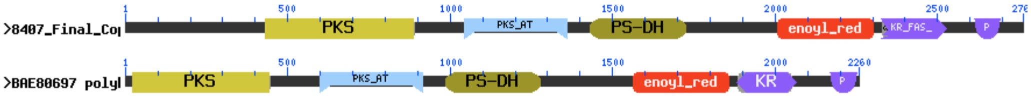

Figure S11. Domain order of PKS8407 and *Alternaria solani* PKSF BAE80697. PKS = ketoacyl synthase, AT= acyl transferase, DH = dehydratase, AdoMet = methyltransferase, enoyl red = enoyl reductase, KR = ketoreductase.

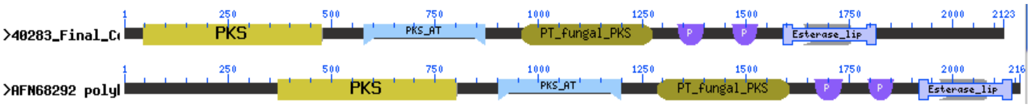

Figure S12. Domain order of PKS40283 and PKSA of *Alternaria alternata* AFN68292. PKS = ketoacyl synthase, AT= acyl Table 14. Domain order of PKS3398 and polyketide synthase of *Metarhizium brunneum* KID62944.

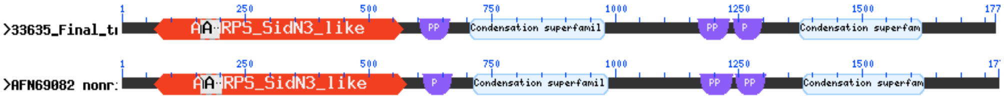

Figure S13. Domain order of NRPS33635 and NPS6 of *Alternaria alternata* AFN69082. A=adenylation, Condensation superfamil = condensation, PP = peptidyl carrier protein?

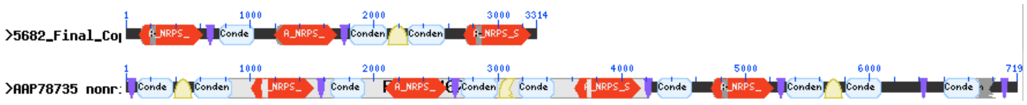

Figure S14. Domain order of PKS5682 and *Alternaria brassicae* nrps1 AAP78735.

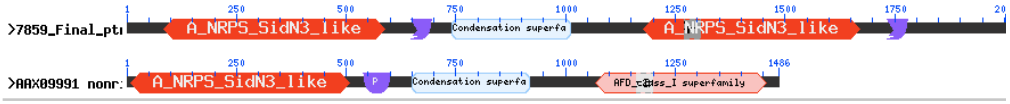

Figure S15. Domain order of NRPS7859 and NPS9 of *Cochliobolus heterostrophus* AAX09991. A=adenylation, Condensation superfamil = condensation, P = peptidyl carrier protein?

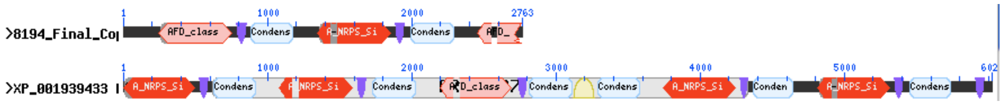

Figure S16. Domain order of NRPS8194 and HC-toxin synthetase of *Pyrenophora tritici-repentis*.

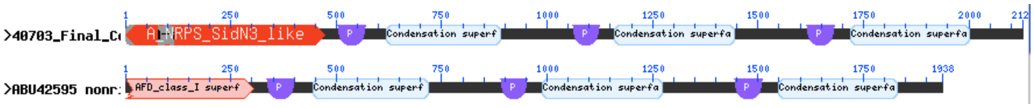

Figure S17. Domain order of NRPS40703 and NPS2 of *Alternaria brassicicola*.

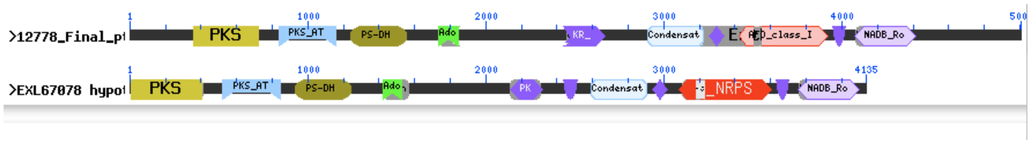

Figure S18. Domain order of PKS12778 and hypothetical protein from *Fusarium oxysporum f. sp. conglutinans* EXL67078. PKS = ketoacyl synthase, AT= acyl transferase, DH = dehydratase, AdoMet = methyltransferase, KR = ketoreductase, Condensat = condensation, A NRPS = adenylation, NADB Ro = NAD binding Rossman folding.

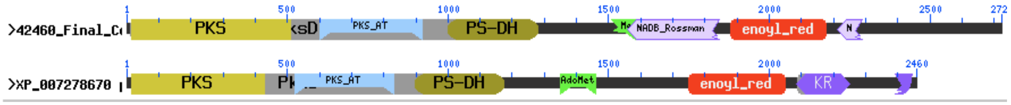

Figure S19. Domain order of PKS42460 and hypothetical polyketide synthase of *Colletotrichum gloeosporioides* XP_007270670. Enoyl red = enoyl reductase .

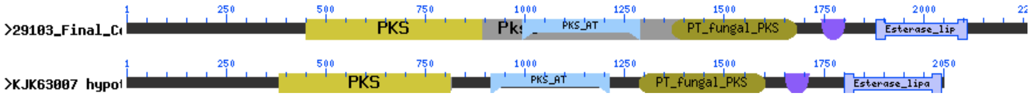

Figure S20. Domain order of PKS29103 and hypothetical protein of *Aspergillus parasiticus* KJK63007.

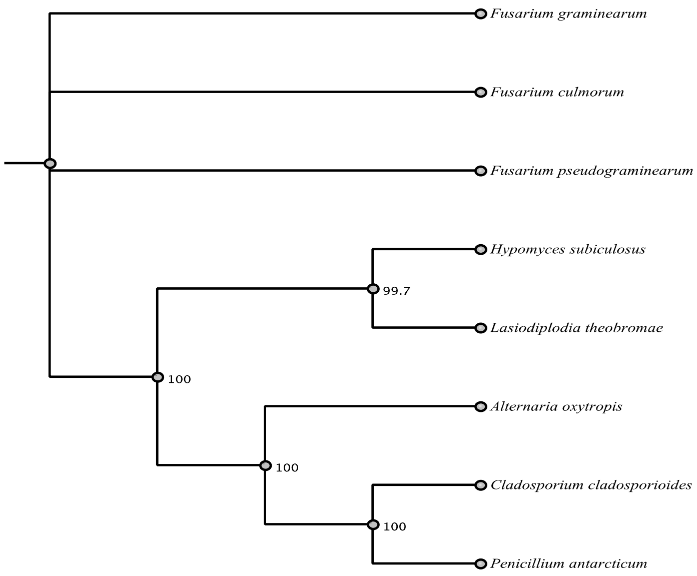

Figure S21. Maximum parsimony tree (MUSCLE alignment and 1000 replicates). *Lasiodiplodia theobromae* was used as the outgroup. Fungi in tree are pblast results of 17612 sequence.

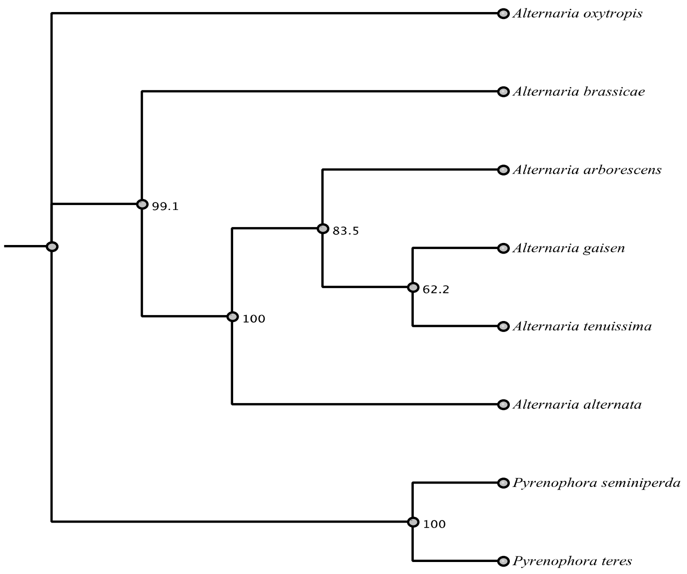

Figure S22. Maximum parsimony tree (MUSCLE alignment and 1000 replicates). *Pyrenophora teres* was used as the out-group. Fungi in tree are pblast results of 5682 sequence.

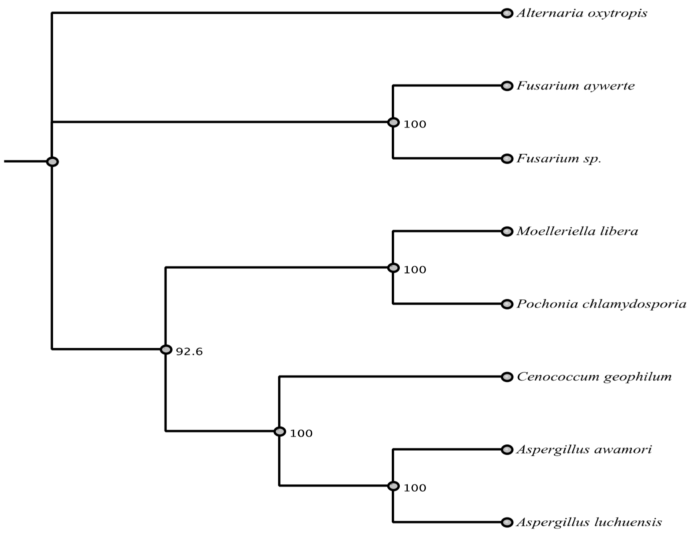

Figure S23. Maximum parsimony tree (MUSCLE alignment and 1000 replicates). *Aspergillus luchuensis* was used as the outgroup. Fungi in tree are pblast results of 9132 sequence.

Table S1. GenBank accession numbers and amino acid percent identity of blast results of 2122 sequence. .

Organism	Accession	Amino Acid Percent Identity
Alternaria alternantherae	AOO87092.1	89.59%
Alternaria tenuissima	RYN35181.1	88.97%
Alternaria solani	BAD83684.1	90.13%
Pyrenophora teres	EFQ86427.1	89.01%
Alternaria alternata	XP_018381235.1	89.08%
Alternaria arborescens	RYN41486.1	89.13%
Alternaria gaisen	RYO51295.1	89.09%

Table S2. Genbank accession numbers and amino acid percent identity of blast results of 17612 sequence. .

Organism	Accession	Amino Acid Percent Identity
Penicillium antarcticum	OQD80247.1	64.45%

Cladosporium cladosporioides	A0A0Y0M151.1	62.81%
Hypomyces subiculosus	ACD39758.1	58.65%
Fusarium pseudograminearum	XP_009258258.1	58.00%
Fusarium graminearum	EYB31080.1	57.96%
Lasiodiplodia theobromae	AHV78245.1	56.92%
Fusarium culmorum	PTD11157.1	57.28%

Table S3. Genbank accession numbers and amino acid percent identity of blast results of 8407 sequence. .

Organism	Accession	Amino Acid Percent Identity
Alternaria solani	BAE80697.1	81.14%
Alternaria arborescens	RYO53185.1	80.47%
Alternaria tenuissima	RYN97731.1	80.46%
Alternaria alternata	OWY52567.1	80.50%
Alternaria alternantherae	AOO87095.1	79.93%
Pyrenophora seminiperda	RMZ66475.1	69.35%
Pyrenophora teres	EFQ92739.1	69.54%

Table S4. Genbank accession numbers and amino acid percent identity of blast results of 3398 sequence. .

Organism	Accession	Amino Acid Percent Identity
Lepidopterella palustris	OCK76486.1	74.49%
Metarhizium brunneum	XP_014539917.1	63.54%
Metarhizium robertsii	XP_007819463.1	64.25%
Metarhizium guizhouense	KID81569.1	63.31%
Metarhizium anisopliae	KJK77000.1	64.03%
Aspergillus fischeri	XP_001265535.1	62.66%
Aspergillus terreus	XP_001213896.1	63.00%

Table S5. GenBank accession numbers and amino acid percent identity of blast results of 40283 sequence. .

Organism	Accession	Amino Acid Percent Identity
Alternaria tenuissima	RYN42853.1	95.17%
Alternaria alternata	AFN68292.1	95.21%
Alternaria arborescens	RYO71336.1	95.26%
Alternaria gaisen	RYO46317.1	94.47%
Alternaria alternantherae	AOO87097.1	93.54%
Stemphylium lycopersici	KNG52671.1	90.05%
Bipolaris sorokiniana	XP_007696213.1	88.93%

Table S6. Genbank accession numbers and amino acid percent identity of blast results of 33635 sequence. .

Organism	Accession	Amino Acid Percent Identity
Alternaria alternata	AFN69082.1	91.40%
Alternaria tenuissima	RYN35257.1	91.46%
Alternaria arborescens	XP_028502413.1	91.63%
Alternaria gaisen	RYO50458.1	91.18%
Stemphylium lycopersici	KNG49863.1	85.82%
Bipolaris oryzae	XP_007684283.1	82.59%
Exserohilum turcica	XP_008020405.1	82.62%

Table S7. Genbank accession numbers and amino acid percent identity of blast results of 5682 sequence. .

Organism	Accession	Amino Acid Percent Identity
Alternaria tenuissima	RYN60358.1	90.75%

Alternaria alternata	XP_018382376.1	90.69%
Alternaria arborescens	RYN26111.1	90.51%
Alternaria gaisen	RYO35725.1	90.23%
Alternaria brassicae	AAP78735.1	89.47%
Pyrenophora teres	EFQ92073.1	80.96%
Pyrenophora seminiperda	RMZ69649.1	79.25%

Table S8. Genbank accession numbers and amino acid percent identity of blast results of 9132 sequence.

**Organism**	**Accession**	**Amino Acid Percent Identity**
Fusarium sp.	RBR08923.1	74.84%
Fusarium aywerte	ALQ32761.1	72.71%
Moelleriella libera	KZZ87545.1	69.00%
Pochonia chlamydosporia	XP_022284243.1	67.00%
Cenococcum geophilum	OCK93825.1	61.71%
Aspergillus luchuensis	OJZ90116.1	58.53%
Aspergillus awamori	GCB20567.1	58.30%
